# Diverse spatial, temporal, and sexual expression of recently duplicated androgen-binding protein genes in *Mus musculus*

**DOI:** 10.1186/1471-2148-5-40

**Published:** 2005-07-14

**Authors:** Christina M Laukaitis, Stephen R Dlouhy, Richard D Emes, Chris P Ponting, Robert C Karn

**Affiliations:** 1 Department of Biological Sciences, Butler University, 4600 Sunset Ave., Indianapolis, Indiana 46208, USA; 2 Internal Medicine Residency Program, St. Vincent Hospital, 2001 W. 86th St., Indianapolis, Indiana 46260, USA; 3 Department of Medical and Molecular Genetics, Indiana University School of Medicine, 875 W. Walnut St., Medical Research and Library Building, Indianapolis, Indiana 46202, USA; 4 MRC Functional Genetics Unit, Department of Human Anatomy and Genetics, University of Oxford, South Parks Road, Oxford OX1 3QX, UK; 5 Department of Biology, University College London, Darwin Building, Gower St., London, WC1E 6BT, UK

## Abstract

**Background:**

The genes for salivary androgen-binding protein (ABP) subunits have been evolving rapidly in ancestors of the house mouse *Mus musculus*, as evidenced both by recent and extensive gene duplication and by high ratios of nonsynonymous to synonymous nucleotide substitution rates. This makes ABP an appropriate model system with which to investigate how recent adaptive evolution of paralogous genes results in functional innovation (neofunctionalization).

**Results:**

It was our goal to find evidence for the expression of as many of the *Abp *paralogues in the mouse genome as possible. We observed expression of six *Abpa *paralogues and five *Abpbg *paralogues in ten glands and other organs located predominantly in the head and neck (olfactory lobe of the brain, three salivary glands, lacrimal gland, Harderian gland, vomeronasal organ, and major olfactory epithelium). These *Abp *paralogues differed dramatically in their specific expression in these different glands and in their sexual dimorphism of expression. We also studied the appearance of expression in both late-stage embryos and postnatal animals prior to puberty and found significantly different timing of the onset of expression among the various paralogues.

**Conclusion:**

The multiple changes in the spatial expression profile of these genes resulting in various combinations of expression in glands and other organs in the head and face of the mouse strongly suggest that neofunctionalization of these genes, driven by adaptive evolution, has occurred following duplication. The extensive diversification in expression of this family of proteins provides two lines of evidence for a pheromonal role for ABP: 1) different patterns of *Abpa/Abpbg *expression in different glands; and 2) sexual dimorphism in the expression of the paralogues in a subset of those glands. These expression patterns differ dramatically among various glands that are located almost exclusively in the head and neck, where the sensory organs are located. Since mice are nocturnal, it is expected that they will make extensive use of olfactory as opposed to visual cues. The glands expressing *Abp *paralogues produce secretions (lacrimal and salivary) or detect odors (MOE and VNO) and thus it appears highly likely that ABP proteins play a role in olfactory communication.

## Background

Genome sequences of primates and rodents [1-3] now allow genome-wide investigations of evolutionary rates and selective processes. The aforementioned studies, and earlier ones, show that mammalian protein-coding genes vary dramatically in their rates of sequence divergence [1,3-6] and that amino acid sequence divergence is greatest for secreted proteins whose genes are expressed in few tissues and least for intracellular proteins found widely in all tissues [7]. These generalizations hold for the great majority (~90%; [1]) of genes that are present in single copies in both primate and rodent genomes. The remaining genes have experienced at least one duplication in either one or both of the primate and rodent lineages.

The process of gene duplication provides material for functional diversification. A newly-duplicated gene may subsequently acquire innovative function ("neofunctionalization") or may retain some of its progenitor gene's functional repertoire ("subfunctionalization"), or a combination of both [8-11]. Such duplicated genes are also rapidly diverging in sequence [1,3] and they are substantially over-represented in functions relating to chemosensation, reproduction, host defense and immunity, and toxin metabolism. Innovation within these functional categories therefore can occur by sequence divergence and/or gene duplication. Adaptive evolution however can also act by modifying gene expression profiles, for example by restricting expression to few tissues. Among genes conserved as single orthologous copies among primate or mouse species, expression variation appears to evolve neutrally and approximately linearly with time [12,13].

We were interested in a related question: How rapidly can expression profiles diverge among duplicated genes? For example, Gu [14] showed an increase in expression diversity in genes involved in *Drosophila *development. We wish to ask about diversification during other evolutionary process, to wit rapidly diverging duplicated genes involved in the four functions listed above. Because expression patterns appear to diverge substantially over long time periods, such as since the primate-rodent (Euarchontoglires) common ancestor [15,16], it is necessary to address this question using many related genes that arose only in very recent times. The most closely-related vertebrate genomes available currently are those of mouse and rat; these lineages diverged approximately 12–24 million years ago (Mya) [17,18]. Thus, by comparing these two genomes we may be able to identify a gene family that is extensively expanded in one, but not necessarily in the other genome, with which to investigate recent expression divergence.

Indeed, we recently identified two families of paralogous genes, clustered together in a 1–2 Mb region of *Mus musculus *chromosome 7 that each meet this criterion. We predict that the Euarchontoglires common ancestor, and the rat and mouse common ancestor, each contained a single member of both gene families; these family members were proximally positioned in a tightly linked head-to-head (bidirectional) gene pair [19,20]. In humans and chimpanzees these genes have accumulated disruptive mutations and are thus likely to be nonfunctional pseudogenes. In the rat lineage, two gene pair duplications have given rise to three gene versions in each family. In the mouse lineage, an extraordinary and rapid burst of duplications has generated over a dozen members of each family, some of which harbor inactivating mutations, whereas others are full-length and apparently functional. Because these mouse genes appear to have all arisen via duplication events since the mouse-rat divergence (12–24 Mya), these are the genes we have chosen to investigate to address the issue of expression divergence.

The two families are termed *Abpa *and *Abpb*/*Abpg *genes (encoding ABPα and ABPβ/ABPγ proteins). They are named according to their proteins that were first described in the literature [21,22]. Androgen-binding protein α subunit (ABPα) forms covalently linked heterodimers with either β or γ subunits, and these are secreted into the saliva following expression in the submaxillary gland. ABPα, ABPβ and ABPγ proteins are secretoglobins, a family of secreted proteins [23] that bind lipophilic ligands (for a review, see [24]) and are present in mammals and birds [25,26], but whose roles in cellular and physiological function all remain obscure [27,28].

Much of the previous work on salivary ABP has focused on determining its function [22]; reviewed briefly in [25]. Laboratory tests of female preference for males carrying different genetic variants of salivary ABP have provided evidence that the protein may mediate sexual preference [29,30]. A role in sexual selection is consistent with the evidence for positive selection in the microevolution of the gene (*Abpa*) for the α subunit, which has a different allele fixed in each of three subspecies of *Mus musculus *[31,32]. These *Abpa *alleles show significantly reduced polymorphism both in their exons and introns and high ratios of nonsynonymous to synonymous nucleotide substitutions (K_A_/K_S_) in the coding region [33,34]. Fixation of different alleles of *Abpa *in the subspecies of *Mus musculus *has been proposed to have occurred by selective sweeps [33]. *Abpb *and *Abpg *also have high K_A_/K_S _values, suggesting that their microevolution has also been driven by positive selection [19,20,22].

Here we investigate the expression of 17 predicted *Abpa*, *Abpb *and *Abpg *genes in 23 mouse glands and other organs. We chose organs and glands to obtain as wide a representation of gene expression in the mouse as possible. We specifically included any tissues where expression of other secretoglobins has been identified (Harderian gland, lacrimal gland, lung, kidney, uterus, skin/sebaceous glands, salivary glands and prostate; [25]). In the head we surveyed the brain and (separately) olfactory lobes, parotid glands, sublingual glands, submaxillary glands, lacrimal glands, major olfactory epithelium (MOE), vomeronasal organ (VNO) and Harderian glands; in the body, we tested skin, adrenal gland, heart, spleen, kidney, testis, lung, liver, pancreas, small intestine, bladder, prostate, ovary and uterus. To test for expression similarity or divergence among paralogous genes we sought to identify transcripts in spatially-distinct tissues and from embryonic or adult individuals of different ages and/or sexes. Our results show how adaptive evolution among paralogous genes has led to expression divergence within a relatively short (12–24 My) period of time. We use these expression patterns to develop a model of paralogue neofunctionalization.

## Results

**Figure 1:**
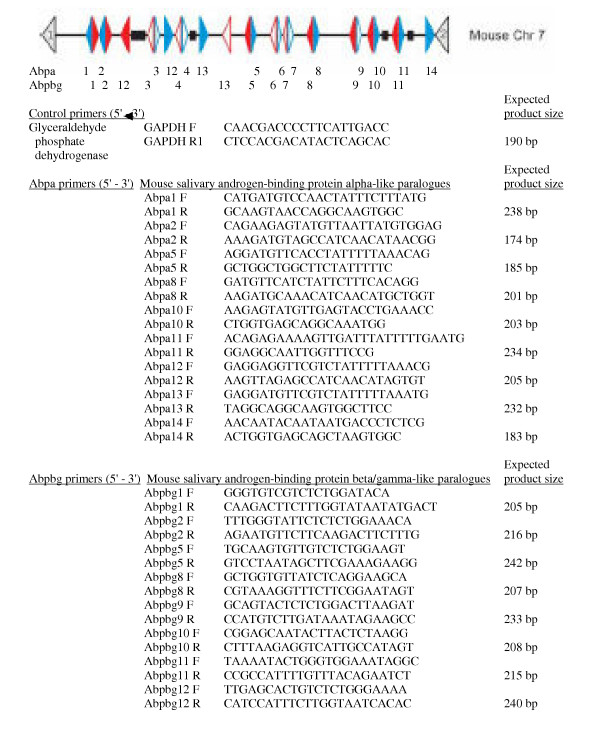
Primer sets used in this study. The order of the *Abpa/Abpbg *paralogues in the mouse genome is shown at the top and the expected size of the PCR product obtained with each primer set is shown to the right of the reverse primer in that set. The 5-to-3 orientations of the genes are shown by the direction of the arrowheads. *Abpa*-like genes are shown in blue and *Abpbg*-like genes in red. Filled arrowheads represent predicted functional genes whereas open arrowheads denote predicted pseudogenes. Gaps (>5 kb) in the genomic assembly of each species are shown as black boxes.

**Figure 2:**
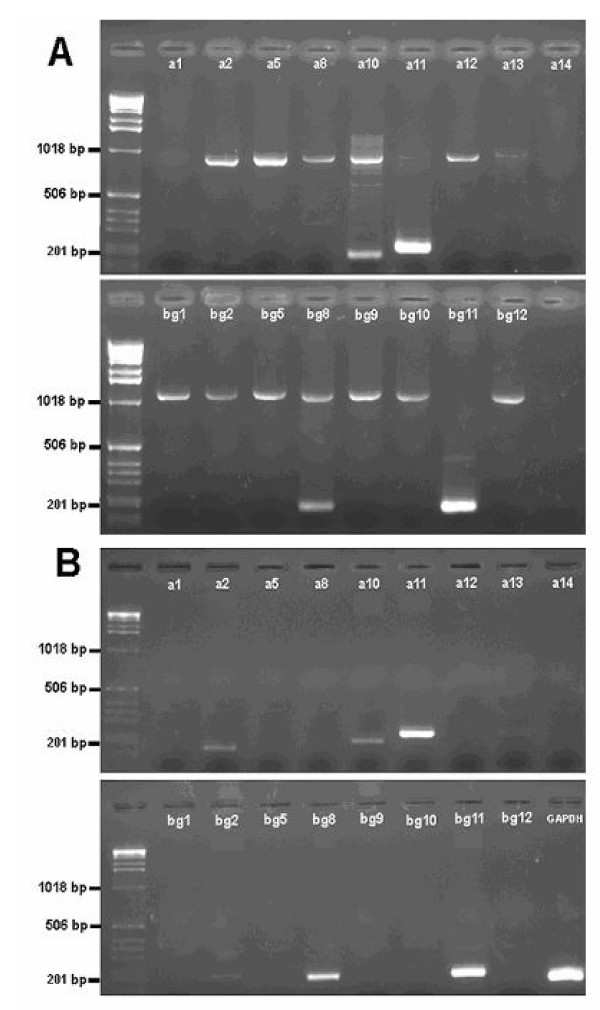
Detection of transcripts in the major olfactory epithelium (MOE) of the mouse. RNA purified from the MOE of C3H/HeJ mice was used to make cDNA, which was used as template in PCR reactions that included, separately, the nine *Abpa *primer sets, the eight *Abpbg *primer sets and the *Gapdh *control primer set. The left-hand lane in all gels contained DNA size markers. Panel A: MOE from a male, where the RNA preparation was used to make cDNA without first being treated with RNase-free DNase. The larger band (>1 kb) that appears in most lanes is the amplification product from genomic DNA that contaminated the RNA preparation. Panel B: MOE from a female, where the RNA preparation was treated with RNase-free DNase before it was used to make cDNA. No genomic amplification products appear on this gel.

Our goal was to survey the glands and other organs of male and female, embryonic, juvenile and adult mice for expression of nine *Abpa *and eight *Abpbg *paralogues previously predicted from the mouse genome to be full-length and apparently functional. Hereinafter *Abpa *and *Abpbg *paralogous genes will simply be identified as "*a*" and "*bg*" with numerical suffixes as described previously [19]. We developed primer sets specific for each putative *Abp *gene (Fig. 1), excluding those paralogues that are predicted to be pseudogenes [19]. Forward and reverse primers of each set fall in exons 2 and 3, respectively (see [20], for the structures of the ABPα, β and γ subunit genes) in order that a much larger product (ca. 1–1.2 kb) is obtained when genomic DNA is the template, compared to that obtained from cDNA templates (ca. 200–300 bp). This design allowed us to test the primer sets, using genomic DNA as templates. Each primer pair amplified a product of the expected size from genomic DNA (not shown). In preliminary experiments the RNA extracted from C3H/HeJ tissues and subsequently used to make cDNA was contaminated with genomic DNA (Fig. 2, Panel A). In subsequent experiments we removed the contaminating genomic DNA by RNase-free DNase treatment, as shown in Fig. 2 (Panel B). In all cases where transcripts were detected, they were of the expected sizes. We verified a representative of each paralogue with DNA sequencing.

The quality of the cDNA made from RNA extracts was tested using an internal control, a primer set designed to amplify the housekeeping gene *Gapdh *[35]. Only cDNA that showed the clear presence of a band of the expected size with the *Gapdh *primers was subsequently tested for *Abpa/Abpbg *transcripts (see Fig. 2, Panel B for an example of *Gapdh *expression). Because RNA preparations were treated with RNase-free DNase prior to their use as templates in PCR reactions, the product obtained with the *Gapdh *primers could confidently be ascribed to amplification of that transcript, rather than to the amplification of pseudogenes of corresponding size [36].

Although it was not our purpose in this study to precisely quantitate levels of expression of the individual paralogues, we did note reproducible variation in intensity of RT-PCR products. A series of dilutions of a typical template indicated that we could detect expression over a broad range. Most expressing tissues gave a negative result between 10^4 ^and 10^6 ^dilution.

### Detection of transcripts in adult tissues

cDNA from 23 glands and other organs of adult C3H/HeJ mice was tested for the presence of transcripts of nine *Abpa *paralogues and eight *Abpbg *paralogues. As detailed below, we have found evidence of *Abp *expression in 10 of these glands/organs, and we demonstrate that multiple members of these gene families are differentially expressed. Equivalent amounts of RNA were tested for each tissue. All experiments in both male and female mice were repeated using tissue independently isolated from a second animal.

**Figure 3:**
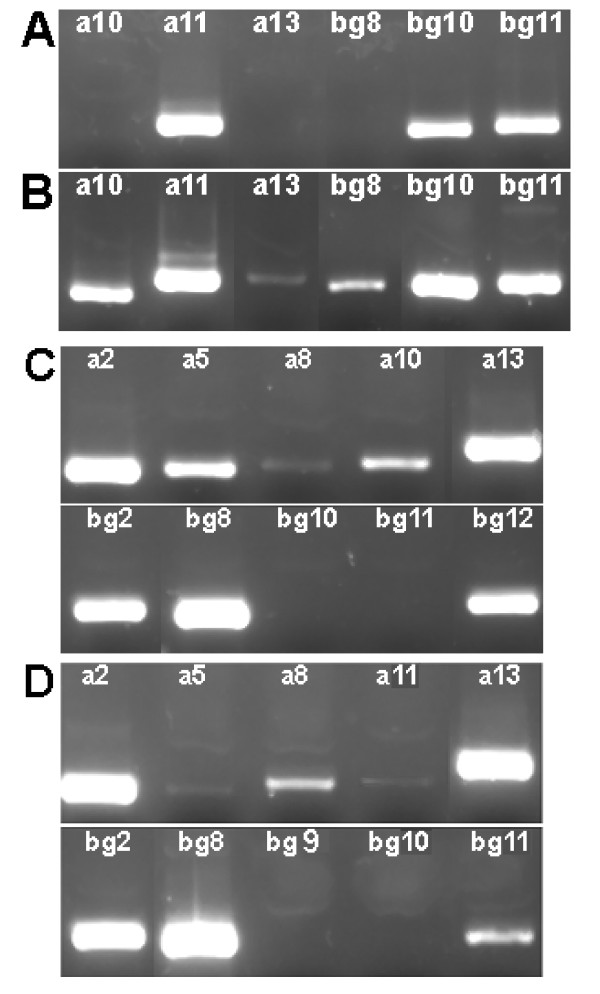
Detection of transcripts in the submaxillary and lacrimal glands of the mouse. Expression testing was done as described in the text and in the legend of Fig. 2. Panel A: Submaxillary gland expression in males; Panel B: Submaxillary gland expression in females; Panel C: Lacrimal gland expression in males; Panel D: Lacrimal gland expression in females.

Figure 2 compares the expression of these paralogues in the major olfactory epithelium (MOE) of adult male (Fig. 2A) and female (Fig. 2B) mice. In both sexes, *a11*, *bg11*, *a10*, and *bg8 *were expressed. As the female also expressed *a2 *and *bg2*, whereas no expression was observed in the male, this represents a likely example of sexual dimorphism of expression. Gene expression in the submaxillary gland is shown in Fig. 3A and 3B. Expression of the *a11, bg11*, and *bg10 *paralogues was found in both male and female submaxillary glands. This was expected from previous findings on the ABPα, β and γ subunit genes, respectively, in the submaxillary gland and its secretions [21,22,37,38]. In addition, the female submaxillary gland exhibits expression of *a10*, *a13 *(weak) and *bg8*, suggesting that sexual dimorphism in *Abp *expression also occurs for this gland.

**Figure 4:**
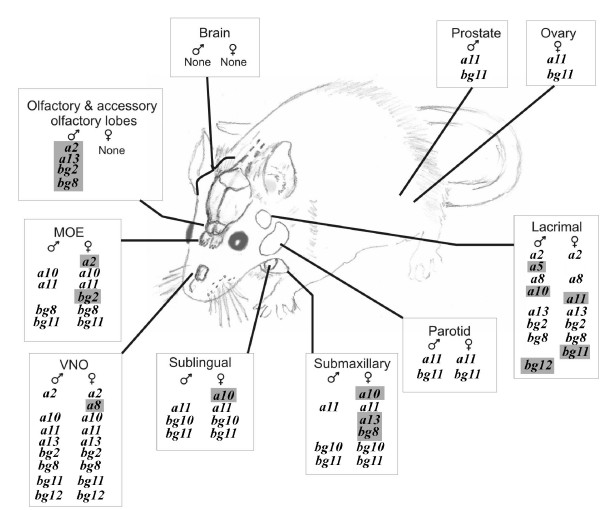
*Abp *paralogue expression shown on a diagram of the glands/organs of a mouse, with expression in males and females differentiated. Paralogues which exhibited sexually dimorphic expression are shaded in grey.

Differential *Abp *expression and sexual dimorphism of *Abp *expression was strikingly apparent in the lacrimal gland where distinct arrays of *Abp *paralogue expressions are seen (Fig. 3C and 3D). By contrast to the MOE and the submaxillary glands, the lacrimal does not show expression of *a11 *or *bg11 *in both sexes. Rather, *a2, a8, a13, bg2 *and *bg8 *are expressed in both sexes, while the male, but not the female, also expresses *a5, a10*, and *bg12*. It is also striking that the female olfactory and accessory olfactory lobes (O/AO combined) did not express *Abp *paralogues, while the male O/AO lobes expressed *a2, a13, bg2 *and *bg8*, as summarized in Fig. 4.

The expression patterns for all the glands and other organs that tested positive for *Abp *expression are summarized in Fig. 4 (the Harderian gland showed only faint bands for a few paralogues in one sex and thus is not included in the figure). We previously suggested that pairs of adjacent *a *and *bg *genes (i.e. those that are identically numbered) might be co-expressed in single tissues or organs [19]. Certainly, the expression of *a2 *and *bg2 *paralogues appears correlated. The most commonly expressed paralogues are *a11 *and *bg11 *and, although their expression is not ubiquitous, in all cases they are co-expressed. In female submaxillary and sublingual glands, *a10 *and *bg10 *are both expressed, along with *a11 *and *bg11*, while only *bg10 *is expressed with *a11 *and *bg11 *in males. The *bg10 *paralogue is co-expressed with *a11 *and *bg11 *only in submaxillary and sublingual glands, but not in parotid glands. The parotid gland data are consistent with other findings [38]. *bg10 *is co-expressed with its genomic partner *a10 *only in the female submaxillary gland. The *a10 *paralogue is expressed in a sexually dimorphic manner in the lacrimal, submaxillary and sublingual glands and in both sexes in the vomeronasal organ (VNO) and the MOE (Fig. 4).

Other paralogues (*a5*, *a8*, *a13*, *bg8*, and *bg12*) are expressed sporadically in various glands. Of these, only *a5 *and *bg8 *are members of *a-bg *pairs in the genome assembly (Fig. 4), but expression of *a5 *only occurs once (male lacrimal), while expression of its partner (*bg5*) was never observed; *bg8 *expression often occurs without that of its partner, *a8*. Notable for the absence of expression in any glands or other organs tested were *a1, a12, a14, bg1, bg5 *and *bg9*; of these, *a12*, *a14 *and *bg9 *are not paired with full-length *a*/*bg *genes, and thus might be pseudogenes despite their full-length coding sequences. On the other hand, *a1 *and *bg1 *and *a5 *and *bg5 *are paired in the genome so there appears to be no absolute rule determining whether paired or unpaired paralogues are expressed.

Thirteen glands and other organs tested negative for *Abp *expression, including brain, skin, adrenal gland, heart, spleen, kidney, testis, lung, liver, pancreas, small intestine, bladder and uterus. With the exception of skin and brain, these are mostly of endodermal and mesodermal origin and are located in the body cavity. By contrast, the glands that show *Abp *paralogue expression are mostly ectodermal in their origins, with the exception of the submaxillary and sublingual glands, which are endodermal.

**Figure 5:**
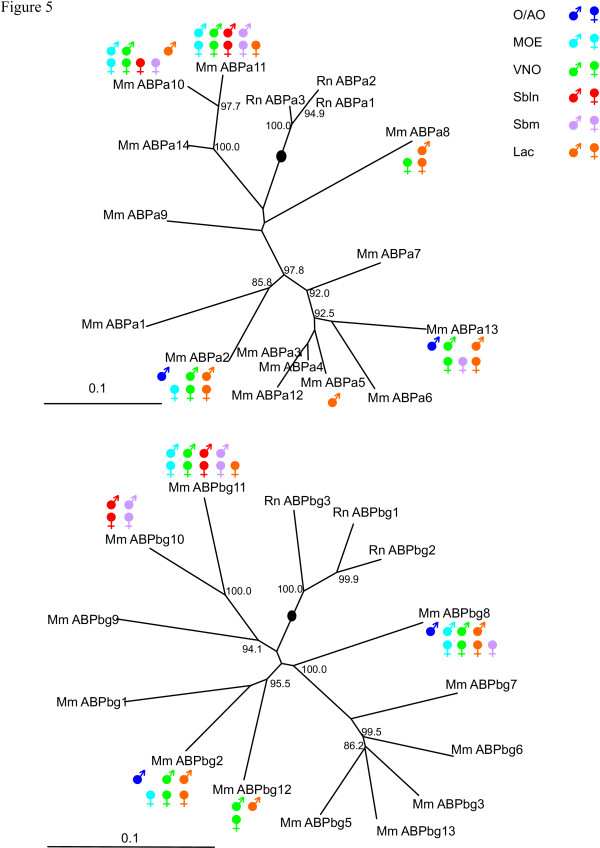
Expressions of *Abp *paralogues painted onto 5' trees which represent phylogenetic relationships among the 5' regions of rodent *Abpa*-like and *Abpbg*-like genes 19. For simplicity, the expression in parotid and prostate/ovary, which is identical in the two sexes (*a11 *and *bg11 *only), has been omitted. The trees were generated with repeat-masked genomic DNA sequences 300 bp and 1 kb, respectively, 5' to the translational start sites of *Abpa *and *Abpbg *genes (see 19 for details). The lineages containing the proposed roots of the trees are shown by black dots and bootstrap values >80% are shown. The key to the expressions in various tissues in the two sexes is shown at the upper right (abbreviations: O/AO = olfactory/accessory olfactory bulbs; MOE = major olfactory epithelium; VNO = vomeronasal organ; Sbln = sublingual gland; Sbm = submaxillary gland; Lac = lacrimal gland).

Figure 5 provides another perspective where the *Abp *expressions for the two sexes are arrayed on phylogenetic trees of the *Abpa *and *Abpbg *paralogues with the intent of discovering possible correlations of expressions of these genes with their evolutionary history. Whilst, this treatment does not reveal any such correlations, it does highlight the sex-limited feature of the expression of many of these *Abp *paralogues.

### Detection of transcripts in embryos and early postnatal stages

**Figure 6:**
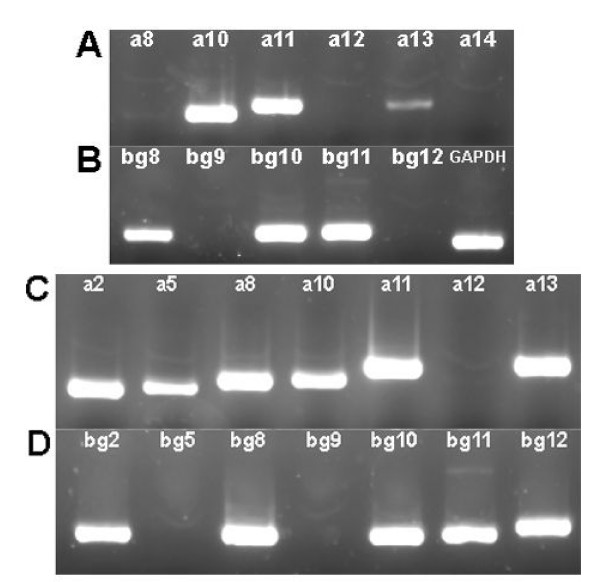
Detection of transcripts in the submaxillary and lacrimal glands of the postnatal day 15 mouse. Expression testing was performed as described in the text and in the legend of Fig. 2. Panels A and B: Submaxillary gland expression of *Abpa *and *Abpbg*, respectively, in males; Panels C and D: Lacrimal gland expression of *Abpa *and *Abpbg*, respectively, in males. Expression in female submaxillary and lacrimal glands was identical to the male glands at this stage of development.

We were interested in determining when the transcripts seen in adult tissues first appear in development. For reference, embryo day 21 (e21) is the day before birth in an average gestation period; the eyes open at day six-seven on average, and the animal goes through puberty at day 30–32 on average in this strain. Therefore we examined embryos at days 13, 17 and 21 of gestation and animals at postnatal days one, six and 15 for expression. RNA was extracted from the entire heads of embryos at 13, 17 and 21 days of gestation, as well as a postnatal day one animal. In the case of days six and 15, we could dissect the combined submaxillary/submandibular region, the brain, the lacrimal glands and the combined MOE/VNO region and for each we were able to generate cDNA. The results for the submaxillary gland and lacrimal gland in the day 15 male are shown in Fig. 6; identical results were observed for the same tissues from day 15 females. The submaxillary gland showed expression of two pairs of paralogues: *a10*/*bg10 *and *a11*/*bg11*, as well as the individual paralogues *bg8 *and *a13*. The lacrimal gland showed expression of four pairs of genes: *a2*/*bg2, a8*/*bg8, a10*/*bg10 *and *a11*/*bg11*, and three unpaired paralogues: *a5*, *a13 *and *bg12*.

**Figure 7:**
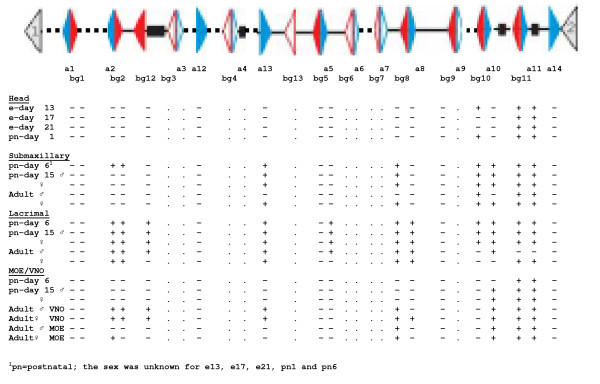
Summary of expression in mouse embryos (e) and early postnatal animals, compared to expression in adults.

The results over the whole developmental time period are shown in Fig. 7, where they are also compared to the expression data observed in the adult tissues. We find that:

1) The *a11*/*bg11 *pair and *bg10 *are expressed in the head between e13 and birth;

2) Immediately following birth (i.e., day one), expression of *bg10*, and *a11*/*bg11 *appears but there is no evidence of any of the other paralogues that eventually are expressed in various tissues in the head (Fig. 5);

3) By day six, however, expression of other paralogues (i.e., in addition to *bg10*, and *a11*/*bg11*) appears in the submaxillary glands (*a2/bg2, bg8, a10*, and *a13*) and the lacrimal glands (*a2/bg2, a5, a8/bg8, a10, a13 *and *bg12*);

4) By contrast, the MOE/VNO region only expresses *a11 *and *bg11 *at day six, and *bg10 *and *a11*/*bg11 *at day 15. By this time point, there is no evidence as yet of paralogues (i.e., *a2/bg2, a13, a8/bg8 *and *bg12*) that are expressed in these tissues in adults;

5) It is also evident that the sexual dimorphism in expression of some of these paralogues has yet to occur by day 15.

## Discussion

The recent sequencing of numerous genomes has made possible comparative studies aimed at enhancing our understanding of gene evolution. It is clear from studies of mammalian genomes that the vast majority of genes have strongly conserved their coding sequences, and generally occur only in single copies [1-3]. Genes involved in adaptation and functional innovation, on the other hand, often show the footprints of positive selection in elevated ratios of nonsynonymous to synonymous nucleotide substitutions rates (K_A_/K_S_; [39]) in their coding regions. In addition, they are subject to frequent duplication, deletion and pseudogene formation [40]. Prevalent amongst rapidly evolving genes are those involved in immunity, reproduction, chemosensation and toxin metabolism [40].

Mouse salivary androgen-binding protein genes *Abpa *and *Abpbg *have evolved under strong positive selection [19,31,33,34,41] and they have been duplicated extensively, mostly in pairs of *Abpa*/*Abpbg *genes, across a 1–2 Mb region of mouse chromosome 7 [19]. This has been a recent expansion peculiar to the mouse, since the rat genome contains only three pairs of *Abpa*/*Abpbg *paralogues, and the human and chimpanzee genomes contain only a single pair whose members are both pseudogenes. These and other observations led to the hypothesis [19] that the common ancestor of mice and rats possessed only a single *Abpa*/*Abpbg *gene pair.

The recently released dog genome contains a single full-length *Abpa/Abpbg *pair, a finding which supports our proposal that this is the ancestral state in eutherian mammals [19]. The cat genome also contains at least one *Abpa*/*Abpbg *pair and these are expressed as the subunits of the Fel dI dimer, the major allergen in the cat [42]. We argue, based on the evolutionary distances (*K*_*S *_values) of the genes, that the alpha and beta/gamma-like genes of cat Fel dI are likely to be the orthologues of the *Abpa *and *Abpbg *genes. The median *K*_*S *_values between rodent *Abpa *or *Abpbg *genes and cat Fel dI are 1.25 and 0.87, respectively which are consistent with the reported substitution rate between rodent and cat orthologous sequence of 0.60 [43] given that neutral rates typically vary within the genome by up to 3 fold. Since the cat orthologue, like a number of *Abpabg *orthologues, is expressed mainly in salivary glands [29,42] it is possible that the original function of the ancestral *Abpa/Abpbg *genes involves secretion from salivary glands and subsequent application to the pelt of the animal.

The large cluster of mouse *Abp *paralogues qualifies in three respects as genes that are rapidly evolving under strong positive selection, namely elevated K_A_/K_S _and a fast duplication rate, and now data showing rapid diversification of gene expression following duplication. The question is which of the four most likely adaptive functions best fits our current understanding of ABP: immunity, reproduction, chemosensation or toxin metabolism? Karn and Dlouhy [32] showed that salivary ABP is ubiquitous in Old World and New World rodents, regardless of their diets, and noted that ABP binds androgens specifically and with relatively high affinity (see also [44] and [41]). They speculated that the function of ABP must be one general to rodents, such as mate recognition, rather than a diet-specific one, such as toxin metabolism. The expression patterns we report here also augur against a role in toxin metabolism because we could not find expression of any of the *Abp *paralogues in mouse liver or spleen, where detoxification of toxic metabolites might be expected to occur.

The developmental sequence of expression of these genes provides clues that help further narrow the choices of functional role for ABP. *Abpa/Abpbg *expression begins in the head of the embryo as early as 13 days of gestation, comprised of a relatively simple pattern of *a11/bg11 *and *bg10*. By the first day of life, this has changed little, but by six days many other paralogues are expressed in the submaxillary and lacrimal glands. Expression proceeds to 15 days of life without yet revealing the sexually dimorphic patterns seen in several glands in adult mice. The transition to sexually dimorphic expression following puberty appears incompatible with roles for these in host immune defense.

It is fascinating that the MOE and VNO are the last tissues to achieve an adult expression pattern. Unlike the lacrimal gland and the salivary glands, these are not secretory tissues. It may be that early expression in the pups' secretory tissues provides signals by which the female parent recognizes her offspring. The pups would not need MOE and VNO expression themselves at this early stage. This distinction hints at a role for ABP in chemosensation, perhaps by participating in detection and recognition of ABP subtypes. Paralogues expressed in the MOE and VNO could be involved in phenotype matching and/or in a system whereby a bound ligand is transferred between paralogues to augment the signal of a proteinaceous pheromone.

Recently, Grus et al. [45] studied V1R genes expressed in the VNOs of the dog, cow and opossum, reporting 8, 32 and 49 intact V1R genes, respectively, in these three species. The numbers of V1R genes in the genomes of mice and rats had been previously reported to be 187 and 102, respectively [46] while the human genome contains about 200 V1R genes, all but 4 of which appear to have been pseudogenized [47,48]. Grus et al. [45] showed a concordance between V1R repertoire size and the complexity of VNO morphology and suggested that these characteristics of the VNO are indicative of the sophistication of pheromone communications within species. These findings are particularly interesting in the context of *Abp *evolution. We previously reported finding a single, pseudogenized *Abp *gene pair in both the human and the chimpanzee genomes, while the genomes of the mouse and rat had approximately 14 and 3 pairs of *Abp *genes, respectively, most of which had full-length ORFs [19]. A single, full-length *Abp *gene pair in each of the dog and cat genomes would be consistent with a pattern of increasing importance of *Abp *genes in conspecific communication, proceeding from potentially no importance in primates to low importance in carnivores to relatively high importance in murid rodents, with the mouse utilizing the most number and diversity of *Abp *gene pairs.

In our recent paper [19], we demonstrated extensive sequence diversification in the numerous *Abpa/Abpbg *paralogues we predicted. In this report, we have shown that *Abp *genes have evolved multiple, often distinct, expression patterns. Once these patterns are mapped to these genes' proposed phylogenetic tree a surprising lack of congruency is observed (Fig. 5): the evolution of gene expression appears not to recapitulate the evolution of coding sequence. Thus it appears that neofunctionalization, rather than neutral drift, has driven the evolution of these genes' expression patterns over relatively short time periods.

The extensive diversification in expression of this family of proteins provides two lines of evidence for a pheromonal role for ABP: 1) different patterns of *Abpa/Abpbg *expression in different glands; and 2) sexual dimorphism in the expression of the paralogues in a subset of those glands. It is clear from our observations that expression patterns of many of the *Abp *paralogues differ dramatically among various glands that are located almost exclusively in the head and neck, where the sensory organs are located. Since mice are nocturnal, it is expected that they will make extensive use of olfactory as opposed to visual cues. The glands expressing *Abp *paralogues produce secretions (lacrimal and salivary) or detect odors (MOE and VNO) and thus it appears highly likely that ABP proteins play a role in olfactory communication. This supports the earlier suggestion, based on behavioral testing, that salivary ABP (the *Abpa *gene, now *Abpa11*) mediates mate preference [29,30]. In the studies of Laukaitis et al., [29], females showed a stronger preference in experiments where the choice was between males tethered to the ends of the test chamber, than they did for the males' territories when the males were absent. Sniffing the face of the male would present the female with much more information (Fig. 4) than the male might leave in his territory in the form of shed skin flakes or hair which had been coated with saliva during grooming [29].

If the variety of proteins produced by expression of the mouse *Abp *paralogues act as components of a complex pheromone system, then the secretions of the lacrimal gland and salivary glands provide a constellation of olfactory information, such as an animal's age and sex. Indeed it has been proposed previously that polymorphism in salivary ABP communicates the species/subspecies of the animal [29-32,49]. Thus we suggest that the strong evidence for involvement of *Abp *paralogous genes in adaptive evolution is consistent with a pheromonal role for their protein products.

Laukaitis et al. [20] proposed that the region between *Abpa *and *Abpb *(*a11 *and *bg11 *in [19] and in this study) could contain a sequence that coordinately regulates their expression. Emes et al. [19] suggested that this was possibly true of the bidirectional 5'-5' paired *Abpa *and *Abpbg *paralogue sets in general. The expression data we report here for *a11 *and *bg11 *is consistent with this idea since we have not seen either expressed independently of the other. However, it does not appear to hold true generally, since we observed independent expression of the members of a number of other pairs (e.g., *bg10 *in submaxillary and sublingual glands; *a10 *in VNO and MOE, etc.). Thus, regulation of the expressions of *Abpa *and *Abpbg *members of pairs must be more complex than simple coordinate up- or down-regulation of pairs. The lack of correlation between the evolutionary relationships of the *Abpa/Abpbg *paralogues [19] and their expression patterns (summarized in Fig. 5) support this notion, suggesting that sequences regulating expression of these genes have evolved in a manner that did not parallel the evolution of the structural genes. This implies rapid evolution of expression diversity in the *Abpa/Abpbg *paralogues.

Huminiecki and Wolfe [50] studied divergence of transcription profiles of genes in homologous tissues between human and mouse. They focused on loci where recent species-specific gene duplication occurred within one or the other species, allowing them to compare the transcription profiles of young paralogues in one species with a single orthologue in the other. They found that the presence of species-specific gene duplication accelerates the rate of expression divergence and that the recent duplicates are subject to reduced constraints on their protein sequences. In most cases, they observed that multiple changes in the spatial expression profile have occurred and they concluded that the expression in a new tissue suggests neofunctionalization. These results are consistent with those we reported earlier [19] and those that we report here for the recently and extensively duplicated *Abpa/Abpbg *genes in mice. This is perhaps an even more striking example because the single orthologous pair of *Abpa/Abpbg *genes in primates both degenerated into pseudogenes while expression in rodents has expanded into at least 9 tissues from the 2 tissues reported for the cat [42].

## Conclusion

The multiple changes in the spatial expression profile of these genes resulting in various combinations of expression in glands and other organs in the head and face of the mouse strongly suggest that neofunctionalization of these genes, driven by adaptive evolution, has occurred following duplication. Primates represent an interesting contrast insofar as their single *Abpa/Abpbg *gene pair was pseudogenized, suggesting that its function has become dispensable. Many genes involved in olfactory and pheromonal cues have become pseudogenes in primates [40]. Evolutionary and expression information thus both indicate a role for ABP and its paralogues in pheromonal communication among mice and, perhaps in other animals, one that is likely to be less important in the primate line, at least in the great apes.

## Methods

### Materials

The C3H/HeJ inbred strain of mice was purchased from Jackson Laboratory (Bar Harbor, ME). Mice at age 75–80 days were sacrificed by cervical dislocation; tissues and organs were removed and frozen immediately in liquid nitrogen and thereafter stored at -80°. Emes et al. [19] published the sequences of 14 *Abpa *paralogues and 13 *Abpbg *paralogues in mouse strain C57BL/6. Five of the *Abpa *paralogues and five of the *Abpbg *paralogues were predicted to be pseudogenes because of frameshifts and/or termination codons occurring early in their sequences. Primer sets were designed to specific forward and reverse sequences for the remaining nine *Abpa *and eight *Abpbg *paralogues. Primers were designed to avoid cross-hybridization between paralogues' sequences. Primer sets were purchased from Sigma-Genosys (St. Louis, MO). DNA from strain C57BL/6 was obtained from Jackson Laboratory for use in testing the primer sets.

### RNA extraction, cDNA production and polymerase chain reaction (PCR)

RNA was purified from mouse tissues with a Sigma total RNA purification kit (Sigma Biochemicals, St. Louis, MO). Absorbances at 260 and 280 nm were determined and used to calculate yield, purity and concentration of RNA from tissue extractions. In some instances, the RNA was treated with RNase-free DNase (Sigma Biochemicals). Oligo-dT was used to prime first-strand synthesis from 1 μg of total RNA per 20 μl RT reaction essentially as described in [31] using Sigma AMV reverse transcriptase (Sigma Biochemicals). PCR was performed using Biolase (MidWest Scientific, St. Louis, MO) with 1μl of the RT reaction as previously described [31,33], using a 61° annealing temperature and 20 sec denaturing, annealing and extension times for 30 cycles. The products were separated on 2% agarose gels.

### DNA sequencing

Clean-up of PCR templates was performed using QiaQuick PCR clean-up spin columns (Qiagen Incorporated, Valencia, CA) according to the manufacturers protocol (Qiagen Incorporated) and automated sequencing was performed by ACGT, Inc. (Chicago, IL).

## Authors' contributions

This work arose from a comparative genomic analysis in which RDE, RCK, CML and CPP agreed to divide the bioinformatics analyses (published in 2004) and the expression analyses (this paper) between the two laboratories, Ponting's at Oxford University and Karn's at Butler University. SRD directed and participated in the harvesting of the many mouse tissues required for the study and he contributed to the design of the expression detection experiments. CML and RCK carried out the PCR expression analyses. RDE and CPP created the annotated gene trees. All authors made substantive intellectual contributions to this work and all authors read and approved the final manuscript.
